# Crystal-Plasticity-Based Micro-Mechanical Model for Simulating Plastic Deformation of TC4 Alloy

**DOI:** 10.3390/ma18245486

**Published:** 2025-12-05

**Authors:** Huanhuan Chen, Wei Li, Zhengming Qian, Dong Mi, Yangyang Wu, Siqi Zhang, Can Wu, Keke Li, Tiezheng Tang, Dongfeng Li

**Affiliations:** 1 AECC Hunan Aviation Powerplant Research Institute, Zhuzhou 412002, China; 2 Research Institute of Aero-Engine, Beihang University, Beijing 100191, China; 3 School of Science, Harbin Institute of Technology, Shenzhen 518055, China; 4 Experimental and Innovative Practice Education Center, Harbin Institute of Technology, Shenzhen 518055, China

**Keywords:** Ti-6Al-4V, crystal plasticity, finite element, polycrystalline, HCP

## Abstract

Ti-6Al-4V (TC4) alloy is widely used in aerospace and biomedical applications due to its excellent strength-to-weight ratio and corrosion resistance. Its plastic deformation behavior is strongly influenced by its microstructural characteristics, particularly grain size. In this study, a crystal plasticity model incorporating a Hall–Petch relationship was developed to simulate the plastic deformation of TC4, with explicit consideration of the effect of grain size on slip resistance. The model employs a thermally activated flow rule to describe the kinetics of slip systems, enabling accurate prediction of flow stress and strain hardening across different microstructural conditions. The model is calibrated and validated using experimental stress–strain data from uniaxial tensile tests on specimens with varying grain sizes. Simulation results demonstrate that the model successfully captures the grain-size-strengthening effect and predicts the corresponding evolution of local strain heterogeneity. Furthermore, a critical local equivalent plastic strain criterion was established, which effectively predicts the dependence of macroscopic failure strain on grain size. This work provides a physically based computational tool for optimizing TC4 processing parameters and predicting deformation under service conditions.

## 1. Introduction

The Ti-6Al-4V alloy (TC4) is widely used in fields such as aerospace and medicine due to its advantages, namely, low density, high strength, excellent corrosion resistance, and good biocompatibility [1,2,3]. Microstructural features directly affect the mechanical properties of polycrystalline materials, and this knowledge enables better material design. In addition, investigations at the grain level are based on quantitative analysis of stress states at the microscale and anisotropic plastic flow [4].

Micromechanical models based on crystal plasticity constitutive theory have developed into a relatively complete analytical framework. The crystal plasticity finite-element method (CPFEM) captures the physical characteristics of metal deformation and has a more rigorous physical foundation compared to macroscopic phenomenological models [5]. By calibrating the crystal plasticity model through quasi-static tensile tests conducted on specimens with different orientations, Somlo et al. [6] investigated the anisotropic tensile behavior of additively manufactured TC4. Taking into account thermal stress, back stress, isotropic hardening, and temperature-dependent tensile stress, Gupta et al. [7] investigated the fatigue behavior of TC4 under both intraphase and interphase thermomechanical loading conditions. Arcidiacono et al. [8] adopted the Armstrong–Frederick nonlinear hardening formulation and proposed a microstructure-sensitive fatigue initiation criterion for TC4 to predict the locations of fatigue crack initiation. Based on a simulation framework integrating Cellular Automaton, Chen et al. [9] investigated the anisotropic behavior of TC4 fabricated via the laser-engineering net-splinting process.

It is worth noting that the majority of the crystal plasticity models employed, particularly those in the aforementioned works, are based on power-law formulations. Although widely used due to their simplicity and computational efficiency, power-law models have inherent limitations in accurately capturing thermally activated deformation mechanisms, especially under conditions involving varying temperatures and strain rates. In contrast, exponential-type thermally activated models provide a more physically grounded framework for describing slip system kinetics, making them better suited for high-fidelity simulations of temperature- and rate-dependent plasticity [10]. Yin and Umezawa [11] investigated the temperature-dependent dwell fatigue behavior of TC4 using thermally activated models. A representative volume element (RVE) model was established within a rate-dependent crystal plasticity framework to study the strain rate sensitivity of TC4. Liu and Dunne [12] investigated the role of macrozone crystallographic orientation and morphology in the dwell fatigue behavior of TC4 alloy using an exponential-type flow rule. Later, they further explored the microstructural evolution and fatigue failure behavior of TC4 titanium alloy under cold dwell fatigue conditions [13].

Compared to microstructural experimental characterization, crystal plasticity modeling is better able to isolate and control individual microstructural parameters while offering time-resolved, full-field deformation insights at the slip-system level (which would be experimentally inaccessible), all with significantly reduced costs and time investment [14]. Meanwhile, various constitutive models have also been developed for investigating polycrystalline micro-deformation. However, these models typically employ homogenization assumptions to bridge single-crystal responses with polycrystalline aggregate behavior. For instance, the Taylor model [15] assumes there is uniform strain across all grains, while self-consistent schemes [16,17,18] apply Eshelby’s method [19] to homogenize polycrystals containing ellipsoidal grains. These simplifications limit these methods’ ability to resolve critical microstructural details such as local slip system activation patterns, grain boundary stress concentrations, and deformation incompatibilities between neighboring grains.

Recent advancements in high-resolution experimental techniques such as in situ synchrotron X-ray diffraction and electron backscatter diffraction (EBSD) have enabled direct validation of CPFEM predictions at the grain scale [20,21,22]. These tools allow for more accurate calibration of model parameters and provide valuable insight into intragranular stress evolution and slip system activation. Furthermore, the integration of modeling with advanced experiments facilitates the study of grain boundary effects [23], heterogeneous deformation [24], and the role of crystallographic texture [25]. Thus, physically based CPFEM simulations can be more reliably applied to assess microstructural damage mechanisms.

In this study, we developed a novel thermally activated crystal plasticity framework that uniquely integrates temperature-dependent slip resistance with grain size effects through a Hall–Petch relationship. The proposed model enables accurate prediction of deformation behavior under varying thermomechanical conditions. Systematic experimental validation established quantitative correlations between grain size and strain localization, demonstrating the model’s capability to predict damage initiation in different microstructures. Comparative analysis revealed that the crystal plasticity simulations using EBSD-derived microstructures achieve high predictive accuracy in capturing the macroscopic stress–strain response when validated against experimental tensile curves. The integrated experimental and computational approach presented in this work advances our fundamental understanding of microstructure–property relationships in TC4 while providing a practical tool for deformation prediction in engineering applications.

## 2. Experimental Procedures

Ti-6Al-4V (TC4) alloy specimens were extracted from mechanical components with an initial diameter of 6 mm and a length of 14 mm. The specimens were supplied by AECC Hunan Aviation Powerplant Research Institute, Zhuzhou, China. The chemical composition of the material is presented in Table 1.

**Figure 1:**
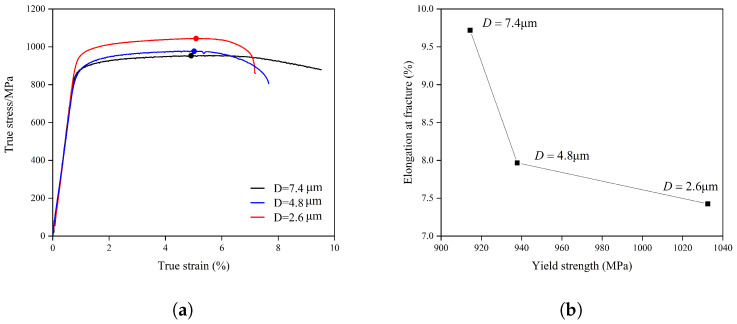
(**a**) Experimental uniaxial tensile true stress–strain curves, with solid circles marking the ultimate tensile strength points. (**b**) Yield strength versus elongation at fracture for different grain sizes.

Uniaxial tensile tests were conducted using a Zwick universal testing machine (100 kN capacity) at room temperature (20 °C) (Figure 1a). The tests were performed at a constant strain rate of 10^−3^ s^−1^. All tensile specimens with different grain sizes were machined from different locations of a single TC4 disk component. Figure 1a shows the true stress–true strain curves, while Figure 1b presents the relationship between yield strength and elongation at fracture.

**Figure 2:**
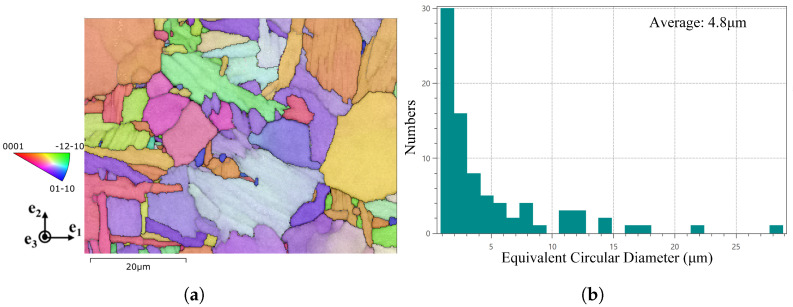
Microstructural of TC4 samples: (**a**) inverse pole figure map and phase distribution determined from electron backscatter diffraction, and (**b**) statistical distribution of equivalent circular diameter for the α-phase grains.

Electron backscatter diffraction (EBSD) data were acquired with a step size of 0.25 μm at a resolution of 261×197 (Figure 2a). The results indicate that the α phase content exceeded 98%, and thus only the hexagonal close-packed (HCP) α phase was considered in the modeling. Representative samples have average equivalent circular diameters (*D*) of 7.4 μm (ranging from 0.9 to 33.9 μm), 4.8 μm (ranging from 0.9 to 27.8 μm), and 2.6 μm (ranging from 0.7 to 24.9 μm) (Figure 2b shows the *D* = 4.8 μm case).

As shown in Figure 1a, the three true stress–true strain curves reach their maximum true stress at strains of 0.028, 0.030, and 0.033, respectively; these points are marked with solid circles. All true strain values reported are dimensionless. A clear grain-size dependence is observed—the finer the grain size, the larger the strain corresponding to the maximum true stress. This trend suggests that finer-grained microstructures can accommodate greater uniform plastic deformation prior to the onset of damage, whereas coarser grains exhibit earlier strain localization and reduced strain-hardening capability.

## 3. Micromechanical Model for Polycrystals

Polycrystal models utilizing the finite-element method enable the discrete representation of grain morphology and continuum-scale material deformation while explicitly accounting for intergranular interactions to predict the aggregate behavior of polycrystalline materials. When subjected to macroscopic loading conditions, individual grains undergo complex deformation modes dictated by mechanical constraints imposed by their local grain neighborhood. Under the influence of specific macroscopic deformation paths, certain grains may exhibit pronounced strain localization arising from incompatibility with adjacent grains. Such localized finite deformations can induce substantial lattice distortions, thereby modifying crystallographic orientation distributions (texture evolution) and influencing subsequent mechanical behavior through geometric effects. Thus, the incorporation of finite-strain single-crystal constitutive relations becomes essential for accurate microscale representation within polycrystalline simulations.

### 3.1. Kinetic Formulation for a Single Crystal

The mechanical deformation of crystalline materials is typically described through a combination of elastic lattice distortions, crystalline rotation, and dislocation-induced slip across multiple slip systems [26,27]. A general description of plastic deformation starts typically from the multiplicative decomposition of the deformation gradient as proposed by Lee [28]:

(1) $$F=F^{e}F^{p},$$

where $F^{e}$ is the elastic part of the deformation gradient (F), and $F^{p}$ is the plastic component. The constitutive relationship between stress and strain can be formulated using the second Piola–Kirchhoff stress tensor $T^{*}$ (defined as $T^{*}=\left|F^{e}\right|{\left(F^{e}\right)}^{-1}\sigma {\left(F^{e}\right)}^{-T}$) and its work-conjugate measure, the Lagrangian Green strain tensor $E^{e}$ (given by $E^{e}=\frac{1}{2}\left[{\left(F^{e}\right)}^{T}F^{e}-I\right]$) as

(2) $$T^{*}=C:E^{e},$$

where C is the fourth-order anisotropic elasticity tensor, and σ is the Cauchy stress tensor. In this formulation, the tensor exponent ${\left(\cdot \right)}^{-T}$ denotes the inverse transpose operation (i.e., $A^{-T}={\left(A^{-1}\right)}^{T}$), and the operator: represents the inner product between two second-order tensors. To represent the plastic flow, the plastic velocity gradient, $L^{p}$, is introduced as

(3) $$L^{p}={\dot{F}}^{p}{\left(F^{p}\right)}^{-1}$$

A superimposed dot denotes the time derivative of the variable. The plastic component of the velocity gradient can be expressed as a linear superposition of slip rates across active crystallographic slip systems [29]:

(4) $$L^{p}=\sum_{\alpha =1}^{N}{\dot{\gamma }}^{\alpha }m^{\alpha }⊗n^{\alpha }$$

where ${\dot{\gamma }}^{\alpha }$ denotes the slip rate, $m^{\alpha }$ and $n^{\alpha }$ are the slip direction and slip plane normal of the slip system, α, and *N* is the total number of slip systems. The operator “⊗” is a tensor product. In this paper, a total of 12 slip systems, including 3 basal slip systems, 3 prismatic slip systems, and 6 pyramidal 〈a〉 slip systems, are taken into account. The specific slip systems and slip directions are listed in Table 2.

The constitutive model employs a thermally activated plasticity formulation originally developed by Busso and McClintock [30]:

(5) $${\dot{\gamma }}^{\alpha }={\dot{\gamma }}_{0}\exp\left(-\frac{F}{k\theta }{\langle 1-{\langle \frac{|\tau^{\alpha }|-S^{\alpha }}{\tau_{0}}\rangle }^{p}\rangle }^{q}\right)\mathrm{sgn}\left(\tau^{\alpha }\right)$$

where θ is the thermodynamic temperature; k is the Boltzmann constant; F represents the Helmholtz activation free energy, defined as the minimum energy required to surmount localized barriers in the absence of external stress; ${\dot{\gamma }}_{0}$, p, and q are the flow rule parameters; and the term $S^{\alpha }$ represents the total slip resistance with respect to dislocation motion. The operator “$\langle x\rangle$” assumes a value of *x* when x>0 and is zero otherwise. $\tau^{\alpha }$ denotes the resolved shear stress defined by

(6) $$\tau^{a}={\left(F^{e}\right)}^{T}F^{e}T^{*}:\left(m^{\alpha }⊗n^{\alpha }\right)$$

$\tau_{0}$ is the lattice friction stress at 0 K. The slip resistance of the slip system $S^{\alpha }$ is governed by

(7) $${\dot{S}}^{\alpha }=\sum_{\beta =1}^{N}h_{s}^{\alpha \beta }\left(\frac{S_{sat}-S^{\beta }}{S_{sat}-S_{0}}\right)\left|{\dot{\gamma }}^{\beta }\right|$$

where $S_{0}$ is the initial slip resistance, $S_{sat}$ is the saturated slip resistance, and $h_{s}^{\alpha \beta }$ is the hardening matrix. In order to capture the grain size effect, a Hall–Petch relation [31,32] is applied for $S_{0}$ as follows:

(8) $$S_{0}=S_{s}+\frac{K}{\sqrt{D}},$$

where $S_{s}$ corresponds to the slip resistance for a sufficiently large grain size, *K* is the Hall–Petch coefficient, and *D* is the average grain size. $h_{s}^{\alpha \beta }$ is defined as follows:

(9) $$h_{s}^{\alpha \beta }=h_{0}\left[\omega_{1}+\left(1-\omega_{2}\right)\delta^{\alpha \beta }\right]$$

where $\delta^{\alpha \beta }$ is Kronecker’s Delta, assuming a value of 1 when α=β and zero otherwise. $h_{0}$ is the hardening constant, and $\omega_{1}$ and $\omega_{2}$ reflect the hardening behavior of the material. In this study, $\omega_{1}=\omega_{2}=1$, which corresponds to Taylor hardening. An effective scalar representation of the combined slip activity in all crystallographic systems is provided by the accumulated equivalent plastic strain:

(10) $${\tilde{\varepsilon }}^{p}=\int_{0}^{t}\sqrt{\frac{2}{3}D^{p}:D^{p}}d\tau$$

where *t* denotes the current time, and τ is the integration variable. $D^{p}$ is the plastic deformation rate tensor given by

(11) $$D^{p}=\frac{1}{2}\left[L^{p}+{\left(L^{p}\right)}^{T}\right]$$

The proposed constitutive model was incorporated into the commercial finite-element software ABAQUS 6.14 through a user-defined material subroutine, utilizing an implicit backward Euler integration scheme. Detailed formulations of the model can be found in Refs. [33,34].

### 3.2. Representative Volume Elements

**Figure 3:**
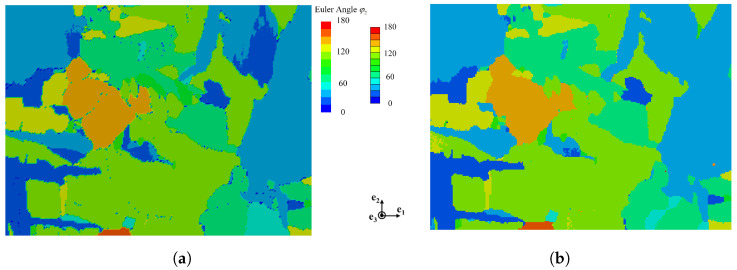
Construction of the representative volume element model: (**a**) experimentally obtained electron backscatter diffraction inverse-pole figure map, and (**b**) corresponding finite-element RVE model with crystallographic orientations assigned from the EBSD data.

Finite-element representative volume element (RVE) models of the microstructure were constructed based on the EBSD (Figure 3a), as shown in Figure 3b. The RVE model applies the grain orientations from the EBSD data for modeling and subsequent calculations.The EBSD-derived microstructure was reconstructed as an RVE with single-element discretization in the out-of-plane direction, maintaining full crystallographic orientation data while enabling efficient computation. In the simulated tests discussed later, the load is applied in the $e_{2}$ direction. The Euler angle $\phi_{2}$ represents the final rotation about the crystal’s *c*-axis. The model employs 43,200 hexahedral elements (C3D8 type), containing 120 grains.

## 4. Results and Discussion

### 4.1. Calibrations of the Material Parameters in the Crystal Plasticity Model

The elastic matrix for HCP can be illustrated as follows:

(12) $$C=\left[\begin{array}{cccccc} C_{11} & C_{12} & C_{13} & 0 & 0 & 0\\ C_{12} & C_{11} & C_{13} & 0 & 0 & 0\\ C_{13} & C_{13} & C_{33} & 0 & 0 & 0\\ 0 & 0 & 0 & C_{44} & 0 & 0\\ 0 & 0 & 0 & 0 & C_{66} & 0\\ 0 & 0 & 0 & 0 & 0 & C_{66} \end{array}\right]$$

where $C_{44}=0.5\left(C_{11}-C_{12}\right)$. The components $C_{ij}$ and the parameters for the CPFEM calibrated from the experimental data are listed in Table 3.

**Figure 4:**
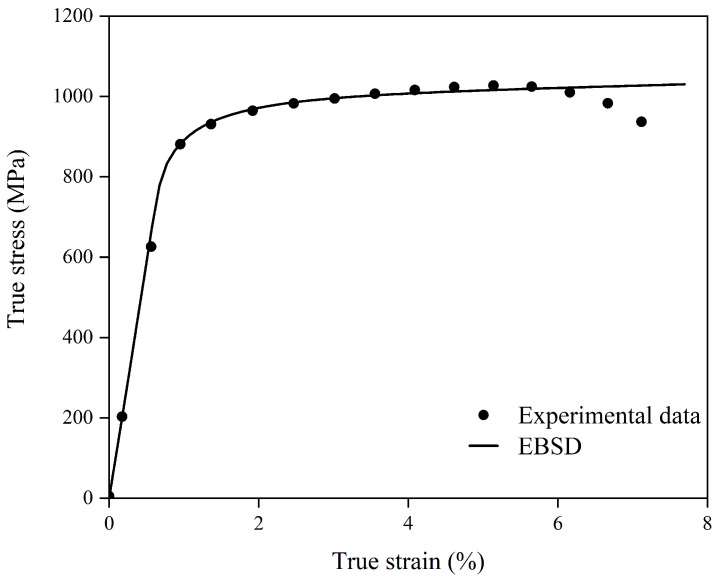
Validation of the crystal plasticity model against experimental data for the sample with an average grain size of 4.8 µm: Comparison between the experimental tensile curve and the simulation results from the EBSD-based representative volume element (RVE) model.

The initial slip resistance for the model whose equivalent circular diameter D = 7.4 μm was determined to be 293 MPa. Figure 4 shows the results simulated by the RVE model.

The numerical results demonstrate that the EBSD-based CPFEM exhibits excellent agreement with the experimental tensile data, albeit with subtle differences in predictive accuracy. It should be noted that the crystal plasticity model employed in this work does not account for damage initiation and subsequent evolution, and thus the comparison with experimental uniaxial tensile data becomes unreliable once the experimental curve reaches the UTS point.

### 4.2. Grain-Size Model Validation

To validate the integrated Hall–Petch formulation within the crystal plasticity framework, the calibrated model was extended to predict the mechanical response of TC4 specimens with finer grain sizes (D = 2.6 μm and 7.4 μm). Using the Hall–Petch coefficient, we calculated the initial slip resistance $S_{0}$ for each grain size: 311 MPa for 2.6 μm and 283 MPa for 7.4 μm.

**Figure 5:**
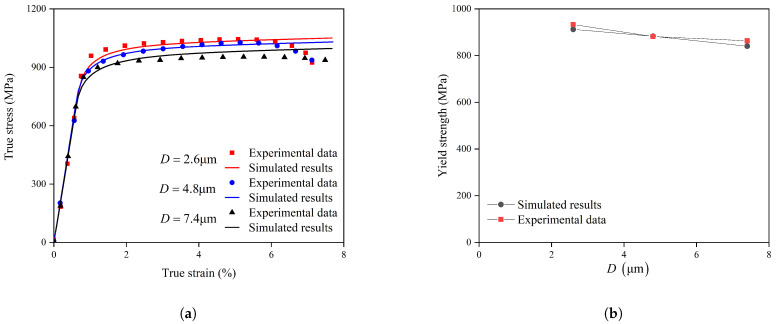
Mechanical response of TC4 alloy with varying grain sizes: (**a**) experimental and simulated true stress–strain curves, and (**b**) Comparison of yield strength between experiments and model predictions as a function of grain size.

Figure 5a compares the simulated stress–strain curves using the derived $S_{0}$ values against experimental data for all three grain sizes. Figure 5b illustrates the yield strength. The CPFEM simulations accurately reproduce the experimental trends across the full range of grain sizes, accurately capturing both the increased yield strength and strain-hardening behavior at reduced grain dimensions. This consistency validates two critical aspects: (1) the physical basis of the thermally activated crystal plasticity model for describing size-dependent plasticity and (2) the appropriateness of the selected Hall–Petch parameters for TC4 alloy.

The numerical results reveal that decreasing grain size from 7.4 μm to 2.6 μm increases yield strength. This agreement confirms that the Hall–Petch relationship, when coupled with the crystal plasticity framework, effectively captures the grain-boundary-strengthening mechanism in TC4. Furthermore, the model correctly predicts the influence of grain size on post-yield behavior—finer microstructures exhibit enhanced strain hardening due to greater dislocation accumulation at grain boundaries.

### 4.3. Effect of Grain Size

**Figure 6:**
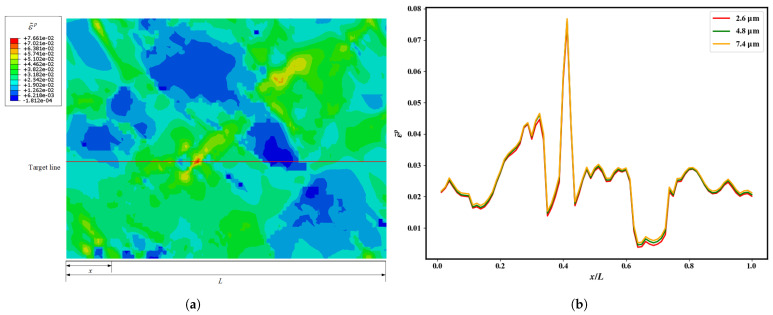
(**a**) Full field distribution of accumulated equivalent plastic strain (PEEQ) at yield across the entire RVE (D = 4.8 µm). (**b**) Local PEEQ values along a target line for different grain sizes, showing the effect of grain size on local strain concentration.

Figure 6a shows the simulated distribution of the accumulated equivalent plastic strain for the alloy with D = 4.8 μm at the yield point (in the RVE (Figure 3b)). A target line was strategically positioned to traverse the region of maximum local cumulative equivalent plastic strain concentration, allowing for quantitative comparison of strain localization patterns across different microstructures (Figure 6b).

Fine-grained specimens exhibited lower cumulative equivalent plastic strain values along the target path, suggesting a more uniform distribution of plastic deformation. In contrast, coarser-grained materials showed more pronounced strain localization, along with higher maximum cumulative equivalent plastic strain values. However, as the grain size increases, the rate of increase in the maximum local cumulative equivalent plastic strain gradually slows, indicating that the extent of strain localization tends to saturate with further grain coarsening.

### 4.4. Failure Prediction

Accurate prediction of failure initiation is crucial for the reliability assessment of metallic components under monotonic loading. While macroscopic tensile tests provide the overall stress–strain response, they lack the ability to directly characterize the local damage accumulation that precedes fracture. Classical criteria, such as the ultimate tensile strength, identify the onset of necking but do not necessarily correspond to the critical local material state for failure. To bridge this gap between global deformation and local failure, we established a novel failure prediction methodology by synergistically integrating experimental data with computational simulations. The core of this approach is to define a critical local failure criterion based on the maximum equivalent plastic strain (PEEQ) at the microstructural level and then calibrate it against the point of maximum true stress measured in uniaxial tensile tests.

The strain at the maximum stress point for the alloy with D = 4.8 μm *ϵ*_*f*_ was identified as 5.01% of the experimental true stress–strain curve in Figure 1a. A finite element simulation of the tensile test for this grain size was conducted up to this macroscopic strain level. The corresponding maximum local PEEQ value in the simulation at this instant was found to be 0.14. This value of 0.14 was subsequently defined as the critical failure criterion ${\tilde{\varepsilon }}_{crit}^{p}$. This criterion was then applied to predict the failure strains for the other grain sizes by determining the macroscopic strain at which the maximum local PEEQ in their respective simulations reached 0.14, i.e.,

(13) $${\tilde{\varepsilon }}_{f}^{p}={\tilde{\varepsilon }}_{crit}^{p}$$

**Figure 7:**
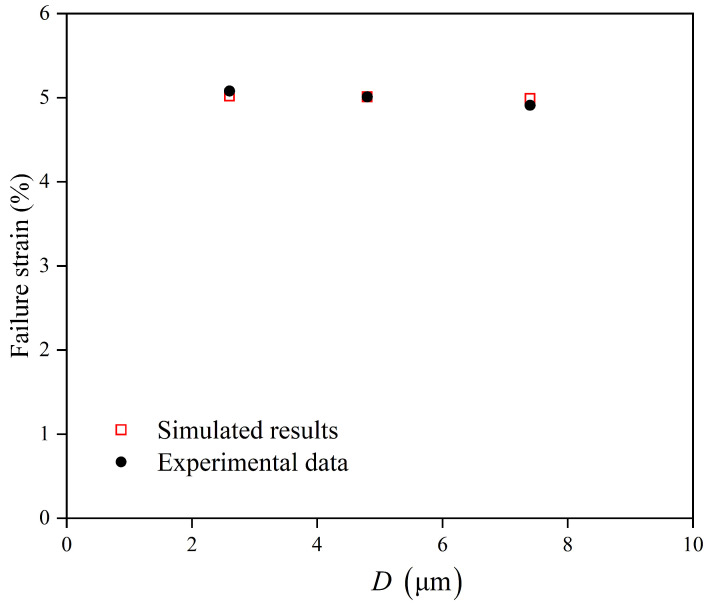
The macroscopic strain required for the local maximum cumulative equivalent plastic strain (PEEQ) to reach the critical value of 5.01%, predicted by finite element simulations for alloys with different grain sizes, and the comparison with the experimental data.

The correlation between the simulated and experimentally measured failure strains across all grain sizes is summarized in Figure 7.

The comparison between the simulation and experimental results demonstrates favorable agreement in predicting the grain size dependence of the failure strain. A clear trend was observed, where the failure strain decreases with increasing grain size. This inverse relationship can be attributed to the effect of grain size governed by strain gradients. In coarser-grained microstructures, stronger constraints at grain boundaries lead to higher concentrations of geometrically necessary dislocations, which accelerate local damage accumulation.

## 5. Summary and Conclusions

This work investigated the plastic deformation and failure behavior of Ti-6Al-4V (TC4) alloy of different grain sizes by integrating uniaxial tensile tests, electron backscatter diffraction (EBSD) characterization, and crystal plasticity finite element simulations. The primary findings and conclusions are as follows:

(1) A thermally activated crystal plasticity model, which explicitly incorporates a Hall–Petch relationship for the initial slip resistance, was successfully developed and calibrated. The model demonstrates high fidelity in predicting the flow stress and strain-hardening behavior of TC4 alloy under uniaxial tension at room temperature.
(2) The model quantitatively captures the grain-size-strengthening effect. Simulations and experiments consistently showed that a reduction in grain size from 7.4 µm to 2.6 µm leads to a significant increase in yield strength, validating the integrated Hall–Petch parameters.
(3) Grain size has a profound influence on strain localization. The numerical results reveal that coarser-grained microstructures develop more intense and heterogeneous local plastic strain concentrations, which act as potential sites for damage initiation.
(4) A local failure initiation criterion, defined by a critical value of accumulated equivalent plastic strain (PEEQ_*crit*_ = 0.14), was established based on the experimental ultimate tensile strength. This criterion successfully predicts the macroscopic failure strain for different grain sizes.
(5) The predicted failure strain decreases with an increasing grain size. This trend is associated with strain localization phenomena that lead to accelerated damage accumulation in coarser-grained microstructures.
(6) It is important to note that the Hall–Petch relationship is typically valid for grain sizes ranging from approximately 1 to 30 µm in titanium alloys. The current model was calibrated and validated within a specific subset (2.6–7.4 µm) of this established range, where its predictive accuracy is high. Extrapolation to grain sizes significantly outside this validated range (particularly to ultra-fine grains < 1 µm or very coarse grains > 30 µm) may require the incorporation of additional physical mechanisms into the constitutive model.

## Figures and Tables

**Table 1:** Chemical composition of TC4 alloy (wt.%).

Element	Al	V	Fe	O	C	N	Ti
Content	5.5–6.5	3.5–4.5	≤0.25	≤0.20	≤0.08	≤0.05	Bal.

**Table 2:** Slip systems in HCP crystals.

	Slip Systems	Slip Plane	Slip Direction
Basal	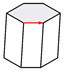	{0001}	$\langle 11\bar{2}0\rangle$
		{0001}	$\langle 1\bar{2}10\rangle$
		{0001}	$\langle 2\bar{1}\bar{1}0\rangle$
Prismatic	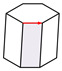	$\left\{1\bar{1}00\right\}$	$\langle 11\bar{2}0\rangle$
		$\left\{10\bar{1}0\right\}$	$\langle 1\bar{2}10\rangle$
		$\left\{01\bar{1}0\right\}$	$\langle 2\bar{1}\bar{1}0\rangle$
Pyramidal 〈a〉	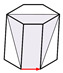	$\left\{10\bar{1}1\right\}$	$\langle \bar{1}2\bar{1}0\rangle$
		$\left\{01\bar{1}1\right\}$	$\langle \bar{2}110\rangle$
		$\left\{\bar{1}101\right\}$	$\langle \bar{1}\bar{1}20\rangle$
		$\left\{\bar{1}011\right\}$	$\langle 1\bar{2}10\rangle$
		$\left\{0\bar{1}11\right\}$	$\langle 2\bar{1}\bar{1}0\rangle$
		$\left\{1\bar{1}01\right\}$	$\langle 11\bar{2}0\rangle$

**Table 3:** Material parameters used in the model developed in this study.

Parameters	Values	Units	Sources
$C_{11}$	162.4	GPa	[35]
$C_{12}$	92	GPa	↓
$C_{13}$	69	GPa	↓
$C_{33}$	180.7	GPa	↓
$C_{66}$	46.7	GPa	↓
*p*	0.45	–	The present work
*q*	1.9	–	↓
*F*	160	kJ/mol	↓
$\tau_{0}$	300	MPa	↓
${\dot{\gamma }}_{0}$	1500	s^−1^	↓
$S_{sat}$	350	MPa	↓
$h_{s}$	100	MPa	↓
$S_{s}$	242.7	MPa	↓
*K*	109.3	MPa$\sqrt{µm}$	[36]

## Data Availability

The original contributions presented in this study are included in the article. Further inquiries can be directed to the corresponding authors.
